# Tubular colonic duplication in an adult patient with long-standing history of constipation and tenesmus

**DOI:** 10.4322/acr.2021.260

**Published:** 2021-05-06

**Authors:** Hisham F. Bahmad, Luis E. Rosario Alvarado, Kiranmayi P. Muddasani, Ana Maria Medina

**Affiliations:** 1 Mount Sinai Medical Center, The Arkadi M. Rywlin M.D. Department of Pathology and Laboratory Medicine, Miami Beach, FL, USA; 2 Mount Sinai Medical Center, Department of General Surgery, Miami Beach, FL, USA; 3 Florida International University, Herbert Wertheim College of Medicine, Miami, FL, USA

**Keywords:** Case Reports, Congenital Abnormalities, Constipation, Diverticulum, Colon

## Abstract

**Background:**

Intestinal duplications are rare congenital developmental anomalies with an incidence of 0.005-0.025% of births. They are usually identified before 2 years of age and commonly affect the foregut or mid-/hindgut. However, it is very uncommon for these anomalies, to arise in the colon or present during adulthood.

**Case presentation:**

Herein, we present a case of a 28-year-old woman with a long-standing history of constipation, tenesmus, and rectal prolapse. Colonoscopy results were normal. An abdominal computed tomography (CT) revealed a diffusely mildly dilated redundant colon, which was prominently stool-filled. The gastrografin enema showed ahaustral mucosal appearance of the sigmoid and descending colon with findings suggestive of tricompartmental pelvic floor prolapse, moderate-size anterior rectocele, and grade 2 sigmoidocele. A laparoscopic exploration was performed, revealing a tubular duplicated colon at the sigmoid level. A sigmoid resection rectopexy was performed. Pathologic examination supported the diagnosis. At 1-month follow-up, the patient was doing well without constipation or rectal prolapse.

**Conclusions:**

Tubular colonic duplications are very rare in adults but should be considered in the differential diagnosis of chronic constipation refractory to medical therapy. Due to the non-specific manifestations of this entity, it is rather challenging to make an accurate diagnosis pre-operatively. Surgery remains the mainstay of treatment. Some reports suggest that carcinomas are more prone to develop in colonic/rectal duplications than in other GI tract duplications.

## INTRODUCTION

Intestinal duplications are rare congenital developmental anomalies with a reported incidence of 0.005-0.025% of births. They commonly affect children below the age of 2.^1^ In more than half of the cases, the ileum and ileocecal valve regions are affected, whereas the colon is less commonly involved (5-15%).^1^^-^^4^ Colonic duplications can be either cystic (80%) or tubular (20%).^5^ While the clinical presentation of such anomalies depends largely on the type and location of the duplication, including abdominal pain, distention, constipation, and other associated complications,^6^^-^^9^ most patients with tubular colonic duplication remain asymptomatic until adulthood.^7^ It has been reported that carcinomas can develop in colonic/rectal duplications,^1^ but no studies have been conducted to quantify this exact risk.

Imaging studies present no substantial aid in ruling in colonic duplications. In most cases, soft tissue mass extrinsic to the bowel wall is the only finding.^7^ Indeed, diagnosis is generally made intra- or postoperatively. While diagnosis can be made by gross examination of the resected bowel segment, key histologic features are usually present and might help in confirming this diagnosis: the presence of three bowel wall layers, Peyer patch-like lymphoid aggregates, and heterotopic gastric, pancreatic, thyroid, or even bronchial epithelium within the intestinal mucosa of the duplication.^1^ The mainstay of treatment is surgical resection to eliminate symptoms.

Herein, we present a case of a 28-year-old woman with tubular duplication of the sigmoid colon. We describe the clinical presentation, gross examination, and microscopic features associated with this entity and the possible mechanisms underlying the development of this anomaly.

## CASE REPORT

**Figure 1:**
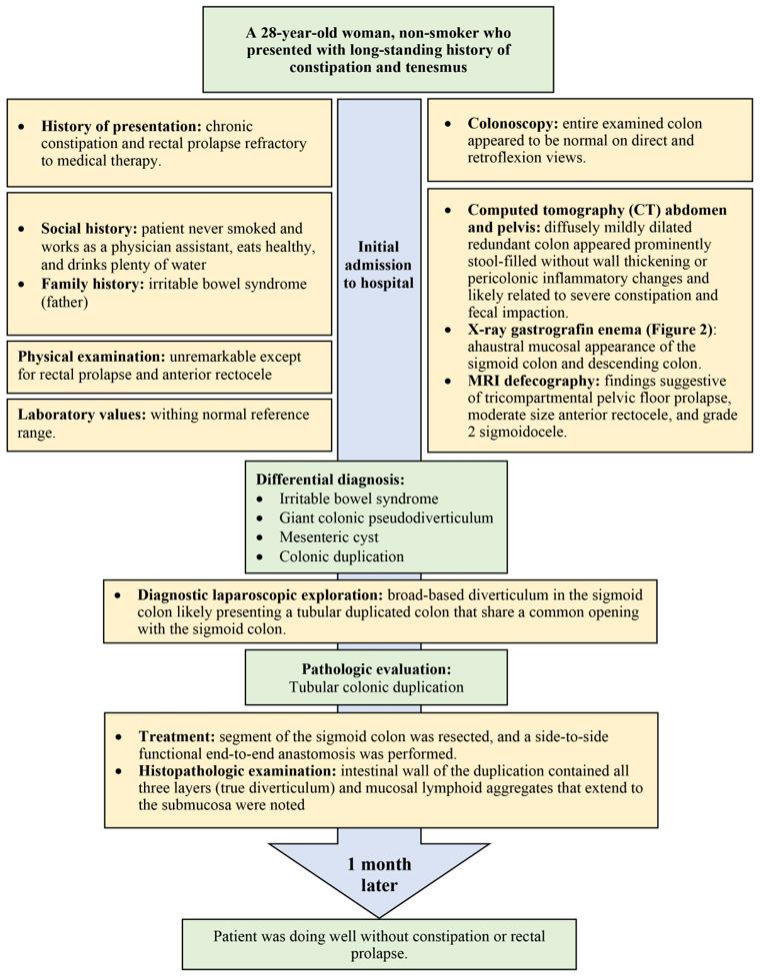
Timeline summarizing major events of the case.

A 28-year-old gravida 0 para 0 (G0P0) woman, non-smoker, presented to our institution for chronic constipation. She had a long-standing history of constipation and tenesmus for more than 3 years. She had used multiple laxatives and linaclotide without much benefit. The patient is a physician assistant and mentioned that she eats healthy and drinks plenty of water. She noticed rectal prolapse on a regular basis when she strains, and occasionally, she had to manually evacuate herself for complete defecation. She had no other reported symptoms. Her symptoms got worse a few months prior to her presentation. She denied any family history of colon cancer or inflammatory bowel disease, but her father has irritable bowel syndrome (IBS). The patient has a surgical history of ovarian cyst removal (Figure 1).

Vital signs were normal, and routine laboratory tests were within the normal reference range. On physical examination, the abdomen was soft, non-distended, and non-tender. Bowel sounds were normal. Yet, obvious rectal prolapse and anterior rectocele were noted. The patient’s BMI was 23.3 kg/m^2^. The patient recently had a colonoscopy at another institution, and results demonstrated an entirely examined colon that appeared to be normal on direct and retroflection views. A year prior, a computed tomography (CT) of the abdomen and pelvis revealed a sigmoid colon which appeared prominently stool-filled and an area suggestive of rectal intussusception without wall thickening or peri-colonic inflammatory changes and likely representing the cause of severe constipation and fecal impaction. At that time, she also had a defecography that showed a 3.5 cm anterior rectocele.

**Figure 2:**
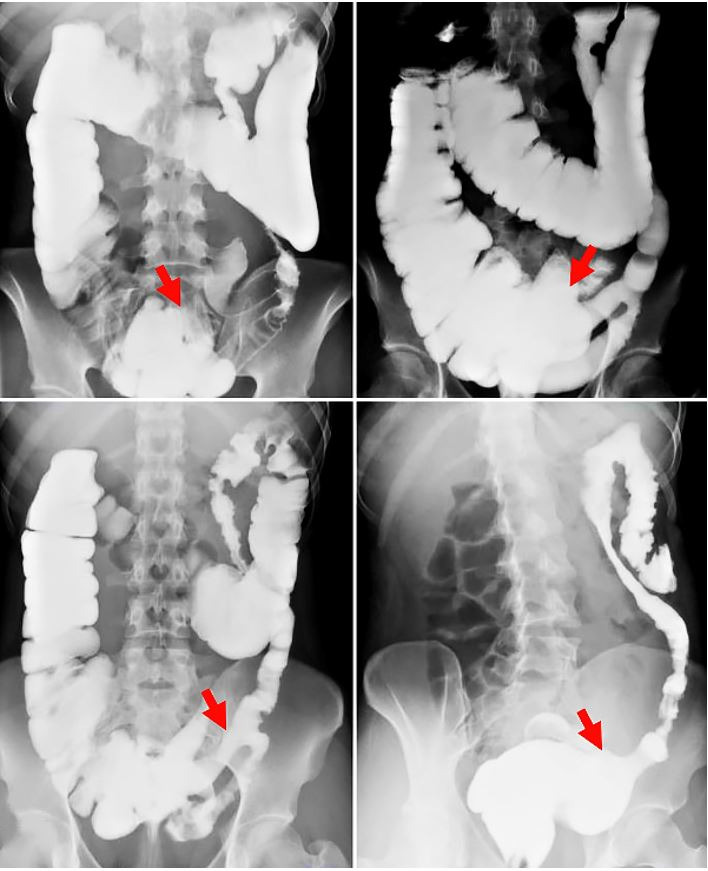
X-ray gastrografin enema. Results showed ahaustral mucosal appearance of the sigmoid and descending colon (red arrows).

**Figure 3:**
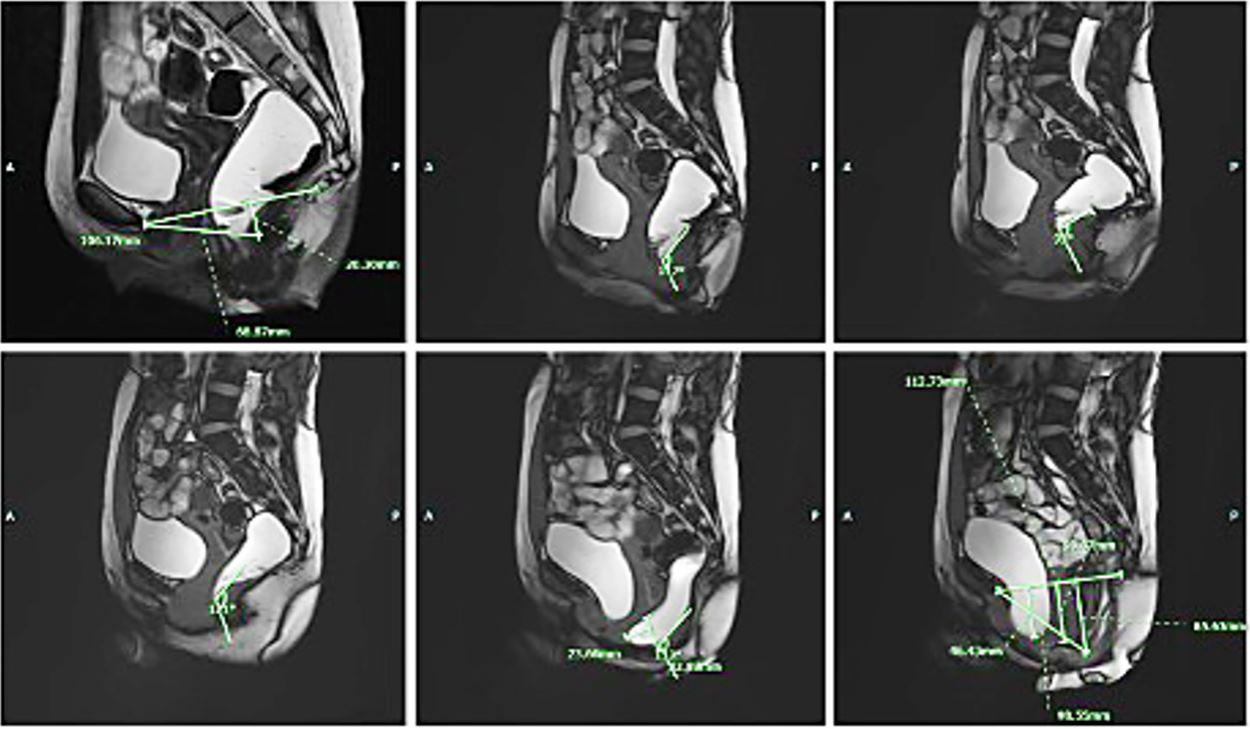
MRI defecography. Findings were suggestive of tricompartmental pelvic floor prolapse, moderate size anterior rectocele, and grade 2 sigmoidocele.

At our institution, an X-ray gastrografin enema was performed, showing ahaustral mucosal appearance of the sigmoid and descending colon (Figure 2). MRI defecography was also done with findings suggestive of tricompartmental pelvic floor prolapse, moderate size anterior rectocele, and grade 2 sigmoidocele (Figure 3).

Those findings can be seen with chronic inflammatory conditions or chronic laxative use. The differential diagnosis included IBS, giant colonic pseudo-diverticulum, mesenteric cyst, colonic neoplasia, or colonic duplication. However, IBS was unlikely since the patient’s symptoms were refractory to medical therapy. Due to the severity of her symptoms and failure of conservative management, the patient agreed to undergo a laparoscopic sigmoidectomy with rectopexy for treatment of her rectal prolapse.

**Figure 4:**
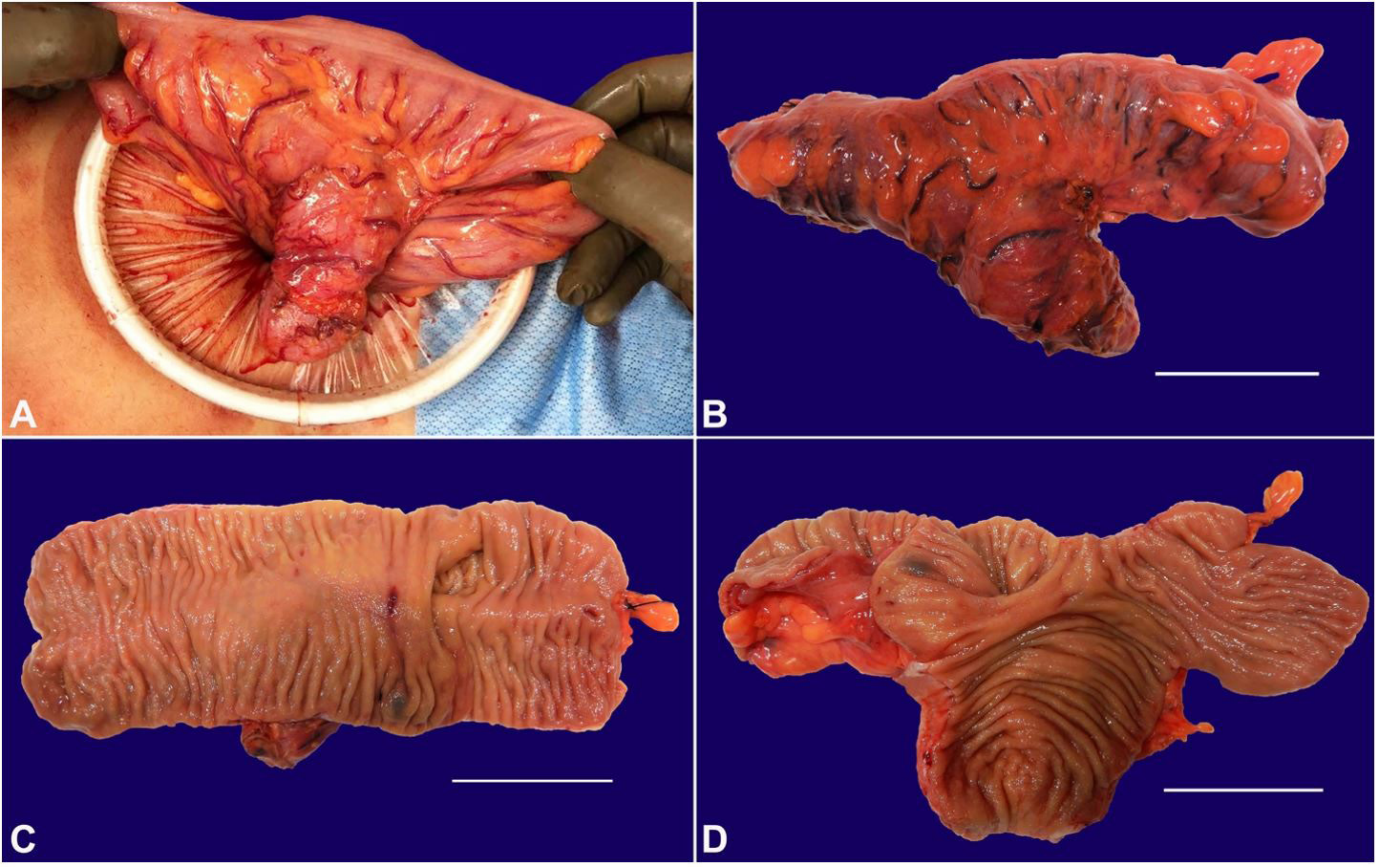
Gross examination of the sigmoid colon. **A –** Broad-based diverticulum is seen in the sigmoid colon likely presenting a tubular duplicated colon that shares a common opening with the sigmoid colon; **B –** Gross pathologic examination of the resected segment of the sigmoid colon shows a longitudinal outpouching located at 4.3 cm away from the closest resection margin, making a 30-degree angle with the sigmoid colon segment, and measuring 7.3 cm in length and 2.6 cm in average diameter (scale bar = 5 cm); **C** and **D –** The colon segment is opened to reveal tan pink mucosa with appropriate colonic mucosal folds in both the sigmoid colon and colonic duplication segments (scale bars = 5 cm).

Diagnostic laparoscopic exploration was performed, revealing a broad-based diverticulum in the sigmoid colon, likely presenting a tubular duplicated colon that shares a common opening with the sigmoid colon (Figure 4).

**Figure 5:**
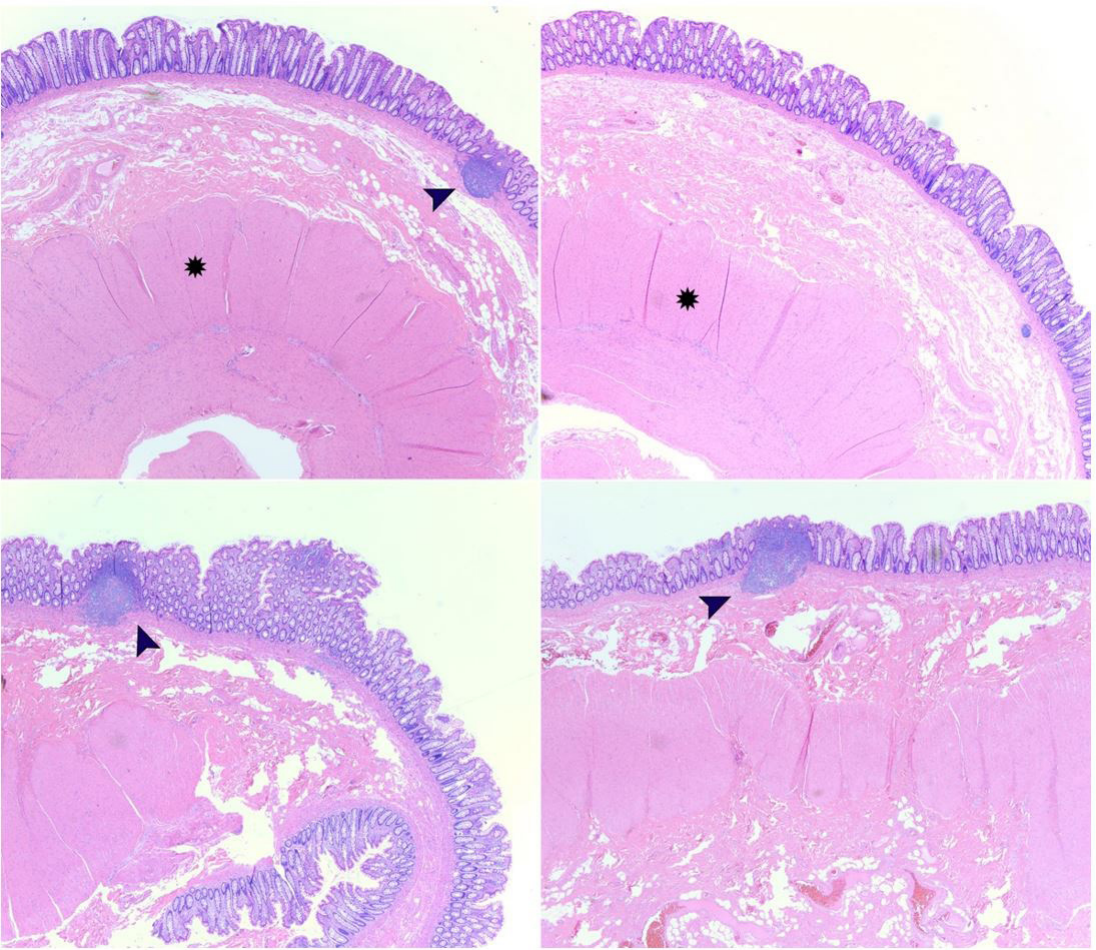
Microscopic examination using H&E stain. Histopathologic examination of the resected tubular colonic duplication demonstrates an intestinal wall containing all three layers (mucosa, submucosa, and serosa; true diverticulum) with well-formed smooth muscle layer (star) and mucosal lymphoid aggregates that extend to the submucosa (black arrows). Microscopic images were examined at 2.5x objective.

A 17 cm segment of the colon was resected, and a side-to-side functional end-to-end anastomosis was performed. The rectum was straightened and secured to the posterior pelvic wall in order to fix the rectal prolapse. On gross pathologic examination, there was an intestinal outpouching located 4.3 cm from one end of the segment of the resected colon, making a 30 degrees angle with the colon. The intestinal outpouching was 7.3 cm in length and 2.6 cm in average circumference; it had a blind end and appeared grossly unremarkable. On microscopic examination, the intestinal wall of the duplication contained all three layers (true diverticulum) with mucosal lymphoid aggregates that extended to the submucosa (Figure 5). The differential diagnosis included giant colonic false diverticulum; however, the smooth muscle layer in that entity is typically not well-formed; hence, gross examination, as well as histopathological features of the resected colon segment, excluded that diagnosis. At 1-month follow-up, the patient was doing well without constipation or rectal prolapse.

## DISCUSSION

Intestinal duplications are rare congenital developmental anomalies.^1^ More than 80% of intestinal duplications are diagnosed in children before the age of 2 years. Anatomically, they are directly contiguous with the segment of the intestine from which they are associated.^10^ Intestinal duplications are “true” duplications where there is a complete or partial formation of a second bowel lumen (doubling) of variable length either communicating with or not with the adjacent native bowel lumen (proximally or distally). In case the duplication is embedded within the bowel wall or the serosa, it is referred to as a duplication cyst.^1^

Various theories have been postulated to contribute to the occurrence of intestinal duplications.^1^ The split notochord theory is based on what has been first described by Bentley and Smith in 1960.^11^ It is a complex congenital malformation that includes vertebral anomalies (spina bifida), central nervous system abnormalities, and intestinal anomalies (fistulas, diverticula, and enteric cysts).^12^ Intestinal duplications could be part of this complex anomaly. Another theory is the failure of normal regression of embryonic diverticula where mesenteric epithelial islands form duplications besides other malrotation abnormalities (omphalocele, Meckel diverticulum, and exstrophy of the bladder).^1^ Intrauterine intestinal ischemia or incomplete recanalization of the enteric tract lumen – which normally occurs at 6-8 weeks gestation – could also lead to the formation of intestinal duplications.^1^ Lastly, if such duplications are associated with another bladder, urethral, or anorectal anomalies, caudal twinning is favored.^13^ The latter prompts the development of caudal duplication syndrome, where structures derived from the embryonic cloaca and notochord are duplicated to various extents.^14^ In our case, since colonic duplication was solely present with no other findings identified on imaging or exploratory laparoscopy, we hypothesize that mesenteric epithelial island could have yielded the development of the tubular colonic duplication due to failure of normal regression of an embryonic diverticulum. However, other causes cannot be ruled out, such as intrauterine intestinal ischemia or incomplete recanalization of the enteric tract lumen at the level of the sigmoid colon.

The ileum and ileocecal valve regions are the most frequently affected sites. (more than half of the cases), while the colon is less commonly involved (5-15%).^1^^-^^4^ From the colonic duplications, transverse colon and cecum appear to be the most common sites of involvement.^6^ Colonic duplications can be either cystic (80%) or tubular (20%).^5^ The rarity of tubular colonic duplications explains why most patients remain asymptomatic until adulthood,^7^ as noted in our patient. Non-communicating duplications usually contain clear alkaline fluid. The gastric mucosa is present in 25% of cases, and acidic fluid is accordingly observed. Ectopic pancreatic tissue has also been reported with present amylase.^3^ Clinical presentation depends on the type and location of the duplication, including colicky abdominal pain, distention, pressure, and other associated complications, such as constipation, tenesmus, intussusception, volvulus, rectal prolapse, ulceration, bleeding, or even malignancy.^6^^-^^9^ In this context, less than 15 cases of malignancy, mainly adenocarcinoma, arising from colonic duplication have been reported in the literature.^15^

No findings are pathognomonic for intestinal duplications on imaging. Work up can begin with plain chest and abdominal X-rays and can be followed by a CT scan. Ultrasonography, barium enema, and colonoscopy can also aid in the diagnosis. Yet, they might not always yield a definitive diagnosis.^16^ Indeed, due to the non-specificity of their results, it is difficult to make a pre-operative diagnosis. In our case, colonoscopy showed that the entire examined colon appeared normal on direct and retroflection views, failing to demonstrate the communicating duplication with the colonic lumen. This might be due to the impaction of the duplication with stool, hindering its visualization on colonoscopy despite it being wide-based. Abdominal CT findings were non-specific, revealing a diffusely mildly dilated redundant colon, which was prominently stool-filled without wall thickening. Moreover, X-ray gastrografin enema demonstrated ahaustral mucosal appearance of the sigmoid and descending colon with findings suggestive of tricompartmental pelvic floor prolapse, moderate size anterior rectocele, and grade 2 sigmoidocele. As noted, none of the imaging studies suggested colonic duplication, making this an unexpected diagnosis in surgery. In most cases reported in the literature, a soft tissue mass extrinsic to the bowel wall is usually identified,^7^ and diagnosis is generally made intra- or post-operatively. Henceforth, the mainstay of management remains elective surgical resection to make the diagnosis, and more importantly, to eliminate symptoms, as we described in the case reported herein. Nevertheless, urgent surgical intervention has been pursued in a number of cases reported in literature where patients presented with massive rectal bleeding, bowel obstruction, or even bowel perforation.^17^

Very few cases of tubular sigmoid duplications in adults have been reported in the literature.^7^ In most cases, patients remain asymptomatic for a long period of time, then they undergo surgery due to life-threatening complications such as bowel obstruction. Roberts et al.^18^ referred to the challenge in making the diagnosis in two cases presenting with abdominal pain and palpable mass on physical examination. Bowel resection was performed in both patients for a suspected carcinoma, but colonic duplication was confirmed on histopathologic examination with no cancer. Al-Jaroof et al.^19^ reported a case of tubular duplication of the sigmoid colon in a 33-year-old woman in which diagnosis was made after exploratory laparotomy, similar to our case. Most of the available literature and most authors recommend surgical resection as the definitive treatment.
